# Curcumin-loaded milk-derived sEVs fused with platelet membrane attenuate endothelial senescence and promote spinal cord injury recovery in diabetic mice

**DOI:** 10.1016/j.mtbio.2025.102036

**Published:** 2025-06-30

**Authors:** Yaozhi He, Siyuan Zou, Jiawei Wang, Wenbin Zhang, Sheng Lu, Mengxian Jia, Yumin Wu, Xiaowu Lin, Ziwei Fan, Qishun Liang, Yizhe Sheng, Qichuan Zhuge, Bi Chen, Minyu Zhu, Honglin Teng

**Affiliations:** a Department of Orthopedics (Spine Surgery), The First Affiliated Hospital of Wenzhou Medical University, Wenzhou, Zhejiang, China; b Department of Emergency Medicine, Second Affiliated Hospital, Zhejiang University School of Medicine, Hangzhou, China; c Department of Thoracic Surgery, Sir Run Run Shaw Hospital, School of Medicine, Zhejiang University, China; d Cixi Biomedical Research Institute, Wenzhou Medical University, Ningbo, Zhejiang, China; e Zhejiang Provincial Key Laboratory of Aging and Neurological Disorder Research, The First Affiliated Hospital of Wenzhou Medical University, Wenzhou, Zhejiang, China

**Keywords:** Diabetes, Spinal cord injury, Milk-derived sEVs, Platelet membrane, Endothelial cell senescence

## Abstract

Spinal cord injury (SCI) causes devastating neurological deficits, and cellular senescence critically contributes to the pathogenesis of various diseases. Notably, endothelial cells (ECs) senescence exerts a pivotal effect on the pathogenesis following SCI. In this study, we found that the number of senescent ECs increased by 18.87% ± 5.91%, and the disruption of the blood-spinal cord barrier (BSCB) was aggravated with an 18% increase in Evans Blue dye extravasation in diabetic mice with spinal cord injury (DM-SCI). To address this pathological process, a bioinspired nanotherapeutic platform utilizing milk-derived small extracellular vesicles with platelet membrane fusion (PM-sEVs) was developed for the targeted delivery of curcumin (Cur). *In vitro*, PM-sEVs-Cur effectively mitigated HG/IL-1β-induced HUVECs senescence by 54.19% ± 5.39% and increased expression of the tight junction protein ZO-1 by 4.33-fold. Mechanistically, Cur attenuated HUVECs senescence by activating the NRF2/HO-1 pathway. *In vivo*, platelet membrane modification enhanced the lesion targeting of sEVs. Treatment with PM-sEVs-Cur attenuated ECs senescence by 34.96% ± 6.59%, preserved BSCB integrity with a 17% reduction in Evans Blue dye extravasation, promoted axonal regeneration with a 6.36-fold increase in neurofilament expression, and improved motor function recovery with an increase of 2.4 ± 0.55 points in Basso Mouse Scale score in DM-SCI. This study highlights PM-sEVs-Cur as a promising therapeutic delivery platform for DM-SCI treatment.

## Graphical abstract

Scheme: Schematic illustration of the manufacturing process of PM-sEVs-Cur and its therapeutic approach in diabetic mice with spinal cord injury.

**Figure:**
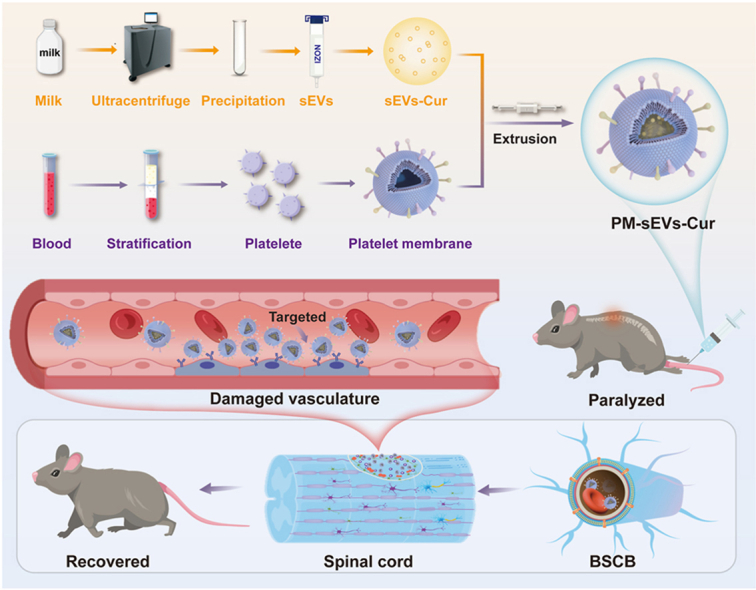

## 1 Introduction

Spinal cord injury (SCI) is a severe medical condition that often leads to permanent motor and sensory dysfunction [1]. With the worldwide prevalence of diabetes, hyperglycemia has emerged as an influential independent risk factor for poor prognosis in many diseases [2]. Evidence indicates that hyperglycemia exacerbates SCI outcomes through multiple pathways, impairing functional recovery in both humans and animal models [[3], [4], [5]]. Notably, hyperglycemia induces premature senescence in ECs, contributing to blood-brain barrier breakdown and subsequent neurological damage [[6], [7], [8]].

Cellular senescence, characterized by irreversible cell cycle arrest, is a critical response to cellular stressors. Although initially protective, the chronic accumulation of senescent cells disrupts tissue homeostasis and impairs repair processes [[9], [10], [11]]. Disruption of the microvasculature and degradation of tight junction (TJ) proteins result in the breakdown of BSCB after SCI, further facilitating immune cell infiltration, chronic inflammation, and the accumulation of senescent ECs [9,[12], [13], [14], [15]]. Due to the particularity of central nervous system (CNS) injury, this long-term and low-grade inflammatory microenvironment is often unfavorable for the regeneration of neuronal axons in SCI [16,17].

Curcumin (Cur), a natural antioxidant with well-documented anti-senescence properties, has shown therapeutic potential across a wide spectrum of disease conditions [[18], [19], [20]]. Studies have demonstrated that Cur inhibits mitochondrial dysfunction and promotes the release and translocation of nuclear factor erythroid 2-related factor 2 (NRF2) into the nucleus. This process regulates cellular antioxidant pathways and enhances cell survivability [21,22]. However, the clinical application of Cur is significantly limited by its poor aqueous solubility, low bioavailability, and rapid systemic clearance, necessitating the development of advanced delivery systems for targeted therapy [23].

Small extracellular vesicles (sEVs), naturally occurring nanoscale membrane particles (50–150 nm) present in biological fluids, have emerged as promising drug delivery vehicles due to their ability to protect and precisely deliver therapeutic cargo [24,25]. While stem cell-derived sEVs show potential in SCI treatment, their clinical translation is hindered by low production yields [26]. Milk-derived sEVs offer a scalable and cost-effective alternative, with the potential for pharmaceutical applications due to their high yield and stability [27]. However, the therapeutic efficacy of unmodified sEVs is limited by their nonspecific accumulation in reticuloendothelial system organs, highlighting the need for innovative targeting strategies in SCI treatment [28].

Recent advances in nanomedicine have introduced cell membrane-coated nanoparticles as a versatile platform combining unique cellular properties [[29], [30], [31]]. Platelet membrane (PM)-coated nanoparticles are particularly promising, as they leverage the natural properties of platelets for immune evasion, subendothelium adhesion, and targeting vascular damage [[32], [33], [34]]. Previous studies have demonstrated that platelet-derived nanovesicles enhance targeting and therapeutic effects in cardiovascular injuries, providing a rationale for their application in SCI [30,35,36].

In this study, we observed an increase in the number of senescent ECs and aggravated BSCB disruption in DM-SCI. *In vitro* experiments revealed that Cur effectively mitigated hyperglycemia/IL-1β (HG/IL-1β)-induced HUVECs senescence through NRF2/HO-1 pathway activation. To overcome the limitations of Cur delivery, we developed a novel nanoparticle system by encapsulating Cur-loaded milk-derived sEVs with platelet membranes (PM-sEVs-Cur). This targeted delivery system successfully localized to the lesion of SCI, improved ECs senescence, preserved BSCB integrity, and promoted axonal regeneration and motor function recovery. This study aimed to: (1) develop PM-sEVs as a targeted delivery platform for DM-SCI; (2) evaluate the ability of PM-sEVs-Cur to localize at SCI lesions and reduce ECs senescence; and (3) assess its therapeutic potential for BSCB preservation and functional recovery in DM-SCI.

## 2 Material and methods

### **2.1** Preparation of sEVs and sEVs-Cur

As previously mentioned, milk-derived sEVs were isolated and purified by going through the steps of centrifugation, ultracentrifugation and size exclusion chromatography [[37], [38], [39]]. Cur was mixed with sEVs at a 1:4 ratio using the freeze-thaw method and incubated for 30 min. Next, the mixture was rapidly frozen at a temperature of −80 °C and then thawed at 37 °C. This cycle was repeated three times. sEVs-Cur was aliquoted and stored at −80 °C for subsequent assays.

### **2.2** Fabrication of PM-sEVs

Platelets were isolated and PM-sEVs were generated as described [34]. Briefly, the platelets were subjected to repeated freeze-thaw cycles. Subsequently, it was centrifuged at 8000 × *g* for 15 min. Thereafter, it was sonicated for 2 min in an FS30D bath sonicator (Fisher Scientific), which operated at a frequency of 42 kHz and power of 100 W. For the fabrication of PM-sEVs, the combinations of PM and sEVs were extruded through 400 nm and 200 nm polycarbonate porous membranes 10 times using an Avanti mini extruder (Avanti Polar Lipids).

### **2.3** Membrane fusion evaluation

Nanoparticle size was measured by NTA using a ZetaView PMX120. To assess the colocalization of PM and sEVs, sEVs were incubated with DiO (Beyotime) for 30 min. Subsequently, the mixture was centrifuged at 100,000 × *g* for 70 min to remove excess dye. PM was incubated with DiL (Beyotime) for 30 min and then centrifuged at 8000 × *g* for 15 min. Fused PM-sEVs were observed by confocal microscope.

### 2.4 *In vitro* stability assays

The concentration of Cur was assessed using a Nanodrop (Thermo Scientific) at 420 nm. Briefly, a standard calibration curve was generated by plotting the concentration of standard Cur versus fluorescent absorbance at 420 nm. At a series of diverse time points, including 0, 30, 60, 90, and 120 min, 100 μL of each sample was taken to determine the Cur concentration.

### **2.5** Cell culture

Human umbilical vein endothelial cells (HUVECs) were cultured at 37 °C in an environment containing 5% CO_2_. To mimic the DM-SCI environment, HUVECs were cultured with HG/IL-1β as described previously [40]. Briefly, HUVECs were initially cultured in ECM (Sciencell, USA, with 5 mM glucose) and transferred to hyperglycemic (25 mM glucose) ECM plus IL-1β (20 ng/mL).

### **2.6** Cellular uptake study

To verify the targeting capability of the PM-sEVs, HUVECs were pre-incubated in an environment with 1% O_2_ for 24 h. Subsequently, they were co-cultured with PBS, DiO-labeled sEVs or PM-sEVs in medium for 1 h. Images were captured using an upright fluorescence microscope (Leica).

### **2.7** EdU assays

The proliferation ability of HUVECs was assessed using the BeyoClick™ EdU Cell Proliferation Kit (Beyotime). The procedure was performed in accordance with the manufacturer's guidelines. Following EdU staining, the cells were observed under an upright fluorescence microscope (Leica). The percentage of EdU^+^ cells was determined by calculating the number of EdU^+^ cells relative to the total cell number.

### 2.8 HUVECs migration

A scratch assay was conducted to evaluate the impact of different treatments on the migration of HUVECs treated with HG/IL-1β. Briefly, HUVECs were seeded in 12-well plates until they reached 100% confluence. Next, a 200 μL pipette tip was used to create scratches. Microscopic images of the scratches were captured immediately and 24 h after wounding. ImageJ was employed to measure the scratch area and calculate the ratio of the scratch area at 24 h to that at 0 h.

### 2.9 Animal and SCI model surgical procedures

Adult C57BL/6 mice (1.5–2M) were obtained from Wenzhou Medical University. All experiments on mice were carried out in accordance with the guidelines on laboratory animal care and use from the Chinese National Institutes of Health. Relevant experimental protocols were administered by the Animal Ethics Committee of the First Affiliated Hospital of Wenzhou Medical University (WYYY-IACUC-AEC-2024-110).

To establish T2DM mice models, C57BL/6 mice were given intraperitoneal injections of streptozocin at a dose of 30 mg/kg/day for 3 days after being fed a high-fat diet for 4 weeks. All mice were subjected to anesthesia (20 mL/kg) through intraperitoneal injection of avertin (2, 2, 2-tribromoethanol). The surgical procedure has been described previously [41]. When the spinal laminae from T8 to T10 were resected, a weight of 10 g was dropped from a height of 5 cm to contuse the spinal cord at T9, resulting in an injury [42,43]. After disinfection and layer-by-layer suturing, the mice were resuscitated on a heating blanket. All mice were randomly assigned to the following groups: Control, Cur, PM-sEVs, sEVs-Cur, PM-sEVs-Cur. After SCI, Cur, PM-sEVs, sEVs-Cur, PM-sEVs-Cur (150 μL of 2 mg/mL) or saline (150 μL) were injected through the tail vein, and the bladder was emptied twice daily manually until the urinating function was restored.

### 2.10 *In vivo* biodistribution study

To assess the targeting efficiency of PM-sEVs in the SCI model, we intravenously injected PBS, PM, sEVs and PM-sEVs. PM, sEVs and PM-sEVs were labeled with DIR (Invitrogen, D12731), a lipophilic red dye. After 24 h of injection, the spinal cord was harvested and examined by an IVIS Spectrum, an *in vivo* imaging system.

### 2.11 Footprint analysis

As mentioned, in order to evaluate the athletic ability, mice ran along a paper-lined runway [44,45]. A blue inkpad was used for the forelimbs and a red inkpad for the hindlimbs. Quantitative analysis was performed on overall stepping ability, stride width, and stride length.

### 2.12 Basso mouse score (BMS)

BMS scoring analysis was performed to examine changes in the locomotive function of mice. Observers who were unaware of the experimental conditions evaluated the mice and assigned scores ranging from 0 to 9 according to the scoring system [46,47].

### 2.13 Western blot (WB)

HUVECs were lysed using RIPA buffer and incubated at 4 °C for 30 min. After extraction with 5x loading buffer, the protein was boiled at 100 °C for 10 min. The samples were separated by SDS-PAGE and transferred onto PVDF membrane. After blocking with skim milk for 1.5 h, the PVDF membrane was incubated overnight with different primary antibodies. The secondary antibodies (1:10,000, Yamei) were used to incubate for 1 h. Detection was carried out using the ECL detection kit (Yamei). Finally, the Quantity One software (Bio-Rad) was used to analyze the images. The primary antibodies included anti-NRF2 (AF0639, Affinity, 1:1000), anti-HO-1 (AF5393, Affinity, 1:1000), anti-P16 (PA5-20379, Invitrogen, 1:1000), anti-P21 (AF6290, Affinity, 1:1000), anti-Occludin (DF7504, Affinity, 1:1000), anti-Claudin-5 (AF5216, Affinity, 1:1000), anti-ZO-1 (AF5145, Affinity, 1:1000), anti-VE-Cadherin (AF6265, Affinity, 1:1000), anti-Integrin β1 (ET1601-17, HUABIO, 1:1000), anti-Integrin α2 (ET1611-57, HUABIO, 1:1000), rabbit anti-GPIbα (DF8519, Affinity, 1:1000), anti-Alix (ET1705-74, HUABIO, 1:1000), anti-TSG101 (ET1701-59, HUABIO, 1:1000) and anti-CD9 (ET1601-9, HUABIO, 1:1000).

### 2.14 Immunostaining

After transcardial perfusion with PBS and then with 4% PFA, the spinal cord was placed in PFA and subsequently transferred to 15% and 30% sucrose solutions until fully immersed. The spinal cord segments were then sectioned to a thickness of 20 μm using a cryostat microtome. For the staining process, the sections were fixed for 30 min with 4% PFA. Next, the sections were subjected to a blocking with 5% BSA and 0.3% Triton X-100 for 1 h. Subsequently, primary antibodies were added, and the sections were incubated overnight at 4 °C. DAPI and appropriate secondary antibodies were added and the sections were incubated for 1 h at room temperature. The following are the primary antibodies: anti-P16 (PA5-20379, Invitrogen, 1:200), anti-ZO-1 (ab221547, Abcam, 1:500), anti-CD31 (AF3628, R&D, 1:200), anti-GFAP (MAB360, Millipore, 1:500), anti-NF (ab8135, Abcam, 1:500), anti-NeuN (E4M5P, CST, 1:400), anti-Tuj1 (66375-1-Ig, Proteintech, 1:400).

For cell staining, HUVECs were fixed with 4% PFA for 30 min. The cells were subjected to blocking for 1 h with 5% BSA and 0.1% Triton X-100. Subsequently, the cells were incubated with primary antibodies overnight. And the DAPI and appropriate secondary antibodies were incubated with cells for 1 h. The primary antibodies used were as follows: anti-γH2AX (ab81299, Abcam, 1:500), anti-P16 (PA5-20379, Invitrogen, 1:200), anti-Claudin-5 (35–2500, Invitrogen, 1:400), anti-ZO-1 (ab221547, Abcam, 1:500).

### 2.15 Senescence-associated β-galactosidase staining (SA-β-gal)

The SA-β-gal staining kit (Solarbio) was used to evaluate the senescence of spinal cord and HUVECs. In summary, the following procedures were performed. The sample was washed once and fixed with fixative for 15 min. Then, 1 mL of dyeing liquid (consisting of 10 μL of fluid A, 10 μL of fluid B, 930 μL of fluid C and 50 μL of X-Gal solution) was added, and the sample was incubated overnight at 37 °C.

### **2.16** Nissl staining

The Nissl staining was performed as previously described [48]. Briefly, spinal cord sections were washed 3 times. Then, they were immersed in cresyl violet for 5 min. Subsequently, they were rinsed in water and followed by 95% ethanol for approximately 3 min. The sections were then incubated in 100% ethanol for 1 min. And they were covered with neutral resins after being immersed in xylene for 5 min.

### 2.17 Hematoxylin-eosin staining (HE)

After washing 3 times, the spinal cord sections were incubated with hematoxylin for 5 min. The samples were then rinsed with water. The sections were stained with eosin for 50 s. After that, they were treated with 95% ethanol and 100% ethanol for 1 min. The sections were made transparent in xylene for 5 min and covered with neutral resins.

### **2.18** BSCB permeability

The permeability of the BSCB was analyzed by evaluating Evans Blue dye (Aladdin) leakage. At 7 days after SCI, 2% Evans Blue dye was administered via intraperitoneal injection. For the qualitative examination of Evans Blue extravasation, two methods were employed. First, images showing overall view changes were captured to compare the spinal cord. Additionally, spinal cords were sliced to a thickness of 20 μm. The fluorescence intensity of Evans Blue in the spinal cord was determined using fluorescence microscopy. Second, N, N-dimethylformamide was used to incubate the spinal cord for 3 days. Subsequently, the samples were centrifuged at 15,000 rcf for 45 min. The detection of Evans Blue was performed using a spectrophotometer with excitation and emission wavelengths of 610 nm and 680 nm respectively [49].

### **2.19** Data analysis

Data were analyzed using GraphPad Prism 8.0 and are presented as mean ± standard deviation (SD). For comparisons between two groups, an unpaired Student's *t*-test was applied. For multiple comparisons, one-way ANOVA with Tukey's post hoc test applied for single-factor experiments (or two-way ANOVA with Bonferroni's test for multiple comparisons, or the Kruskal-Wallis test with Dunn's Multiple Comparison Test for post hoc comparisons) was performed. All statistical evaluations were performed at a 95% confidence interval to determine the significance of intergroup differences. *P* < 0.05 was considered statistically significant. All experiments were independently repeated at least three times.

## 3 Results

### 3.1 Increased markers of cellular senescence and BSCB permeability in DM-SCI

SA-β-gal staining revealed elevated senescence in the DM-Sham group compared to the Sham group by 0.51 ± 0.12 (*p* = 0.011) and by 1.01 ± 0.7 (*p* = 0.0334) in the DM-SCI group compared to the SCI group (Fig. 1A and B). It is interesting to note that a higher proportion of senescent ECs was observed in the DM-Sham group compared with the Sham group by 12.06% ± 4.89% (*p* = 0.0094) through immunofluorescence staining for CD31 and P16. Moreover, this proportion was further increased in the DM-SCI group compared to the SCI group by 18.87% ± 5.91% (*p* = 0.001) (Fig. 1C and D). Additionally, Evans Blue dye extravasation assays showed that the permeability of BSCB in the DM-SCI group was significantly higher than that in the SCI group by 1.14 ± 0.18 (*p* = 0.0085) in the Evans Blue content, 0.63 ± 0.35 (*p* = 0.0085) in lesion area, and 1.87 ± 0.59 (*p* = 0.032) in fluorescence intensity (Fig. 1E–I).

**Fig. 1:**
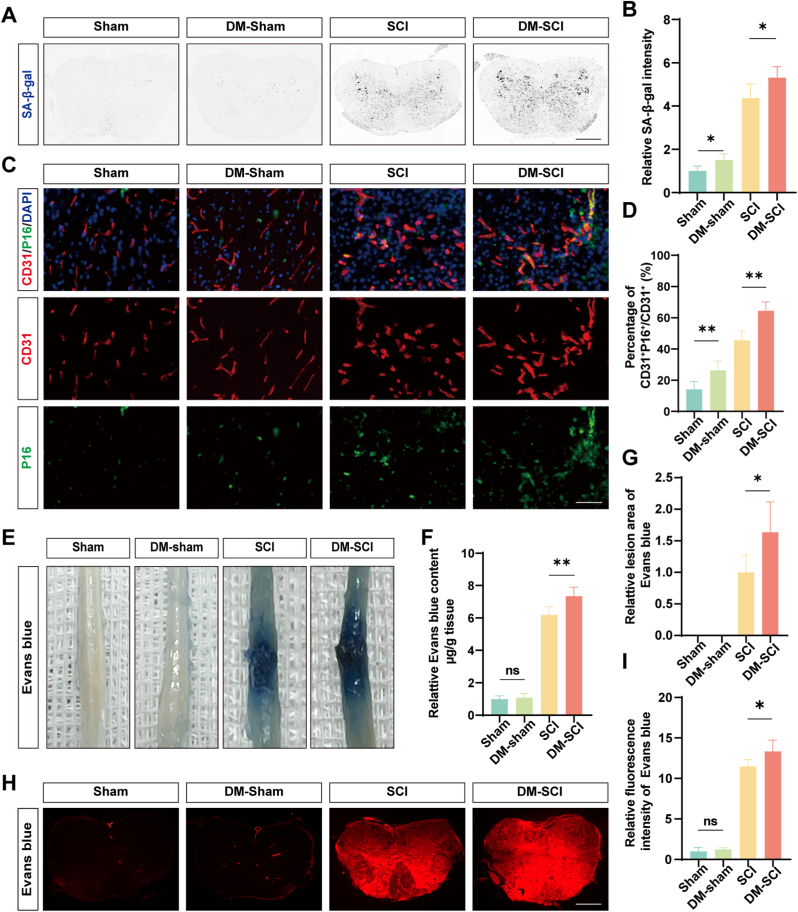
**Increased markers of cellular senescence and BSCB permeability in DM-SCI** (A) Typical images of spinal cord stained with SA-β-gal. (black precipitate indicates senescent cells). The DM-SCI group showed an increase in the number of senescent cells compared to the SCI group. Scale bar = 400 μm. (B) Quantification of the relative intensity of SA-β-gal (n = 5). (C) Typical images of spinal cord stained with CD31 and P16. Merged images show co-localization (yellow), indicating senescent ECs. The DM-SCI group showed an increase in the number of senescent ECs compared to the SCI group. Scale bar = 100 μm. (D) Quantification of the percentage of CD31^+^P16^+^/CD31^+^ cells (n = 5). (E) Typical images from Evans Blue leakage test. Visual inspection revealed more intense and widespread blue staining in the DM-SCI group than in the SCI group, indicating greater Evans Blue leakage. (F) Quantification of the relative Evans Blue content (n = 5). (G) Quantification of relative lesion area (n = 5). (H) Typical red fluorescence images of Evans Blue extravasation. The increased red fluorescence intensity and area in the DM-SCI group compared to the SCI group demonstrated BSCB disruption. Scale bar = 400 μm. (I) Quantification of the relative fluorescence intensity of Evans Blue (n = 5). ∗*P* < 0.05, ∗∗*P* < 0.01; ns indicates not significant. (For interpretation of the references to colour in this figure legend, the reader is referred to the Web version of this article.)

### 3.2 Elevated markers of cellular senescence in HUVECs after HG/IL-1β co-treatment

To replicate the hyperglycemic and inflammatory environments of DM-SCI *in vitro*, HUVECs were exposed to HG/IL-1β co-treatment. Compared to treatment with HG or IL-1β alone, the HG/IL-1β group showed the highest number of senescent cells, with an increase of 36.26% ± 10.53% (*p* < 0.0001) and 33.60% ± 13.48% (*p* < 0.0001) (Fig. 2A and B). Moreover, immunofluorescence staining revealed elevated expression levels of P16 and γH2AX in the HG/IL-1β group. γH2AX intensity increased by 3.05 ± 0.62 (p < 0.0001, HG/IL-1β vs HG) and 2.7 ± 0.77 (p < 0.0001, HG/IL-1β vs IL-1β) (Fig. 2C and D), while P16 intensity increased by 2 ± 1.01 (p = 0.001, HG/IL-1β vs HG) and 1.71 ± 0.59 (p = 0.0047, HG/IL-1β vs IL-1β) (Fig. 2E and F). WB analysis confirmed the upregulation of senescence-associated proteins: P16 expression increased by 1.18 ± 0.33 (p = 0.0003, HG/IL-1β vs HG) and 0.65 ± 0.47 (p = 0.0138, HG/IL-1β vs IL-1β), and P21 increased by 0.58 ± 0.10 (p = 0.0009, HG/IL-1β vs HG) and 0.36 ± 0.12 (p = 0.017, HG/IL-1β vs IL-1β) (Fig. 2I–K). Furthermore, the EdU^+^ cell percentage decreased by 43.81% ± 12.67% (p < 0.0001, HG/IL-1β vs HG) and 31.46% ± 16.06% (p < 0.0001, HG/IL-1β vs IL-1β) (Fig. 2G and H).

**Fig. 2:**
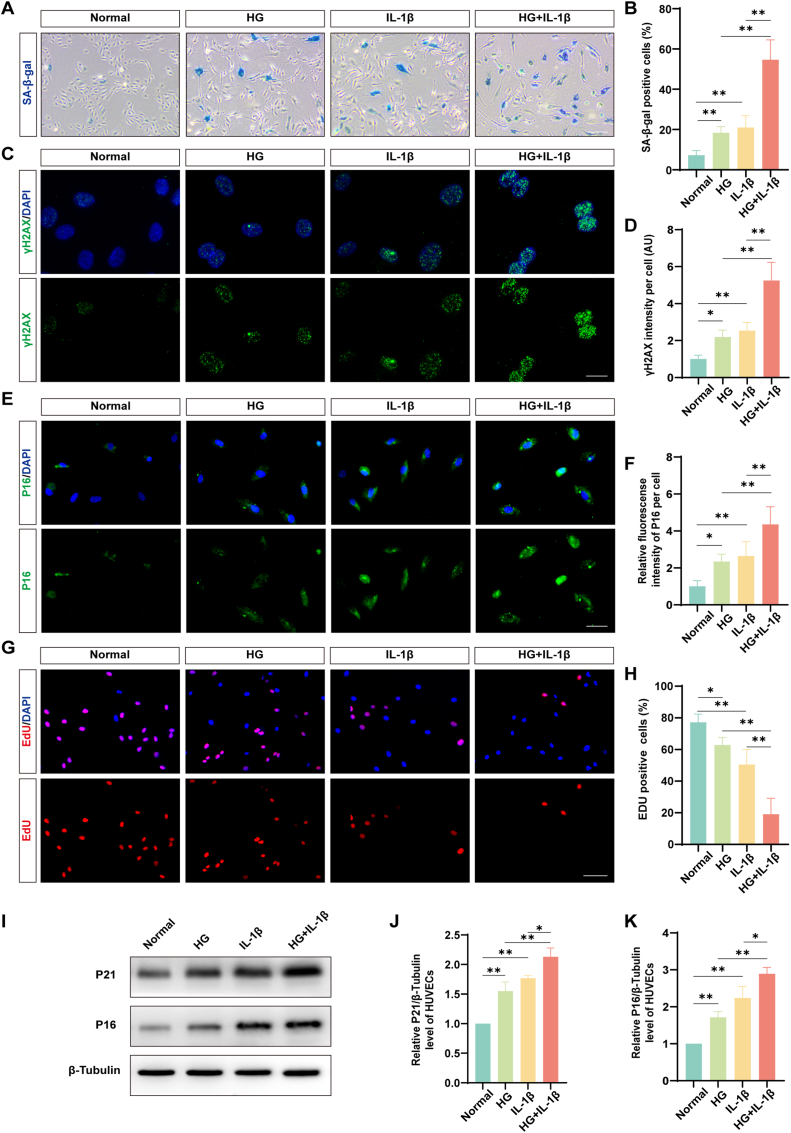
**Elevated markers of cellular senescence in HUVECs after HG/IL-1β co-treatment.** (A) Typical images of HUVECs stained by SA-β-gal (blue stain indicates senescent cells). The number of SA-β-gal^+^ cells increased in the HG/IL-1β group. Scale bar = 200 μm. (B) Quantification of SA-β-gal^+^ cells (n = 5). (C) Typical images of HUVECs stained by γH2AX (green puncta within nuclear, a marker of DNA double-strand breaks). The intensity of γH2AX was increased in the HG/IL-1β group. Scale bar = 20 μm. (D) Quantification of γH2AX intensity per cell (n = 5). (E) Typical images of HUVECs stained by P16 (green, senescence marker). The intensity of P16 increased in the HG/IL-1β group. Scale bar = 50 μm. (F) Quantification of the relative fluorescence intensity of P16 per cell (n = 5). (G) Typical images of HUVECs stained by EdU (red, proliferation marker). The number of EdU^+^ cells was decreased in the HG/IL-1β group. Scale bar = 100 μm. (H) Quantification of EdU^+^ cells (n = 5). (I) Analysis of P21 and P16 expression in HUVECs by WB. The protein levels of P21 and P16 were increased in the HG/IL-1β group. (J) Quantify the expression level of P21 (n = 3). (K) Quantify the expression level of P16 (n = 3). ∗*P* < 0.05, ∗∗*P* < 0.01. (For interpretation of the references to colour in this figure legend, the reader is referred to the Web version of this article.)

### 3.3 Reduction in HG/IL-1β-induced senescence markers and TJ protein degradation in HUVECs by Cur treatment

**Fig. 3:**
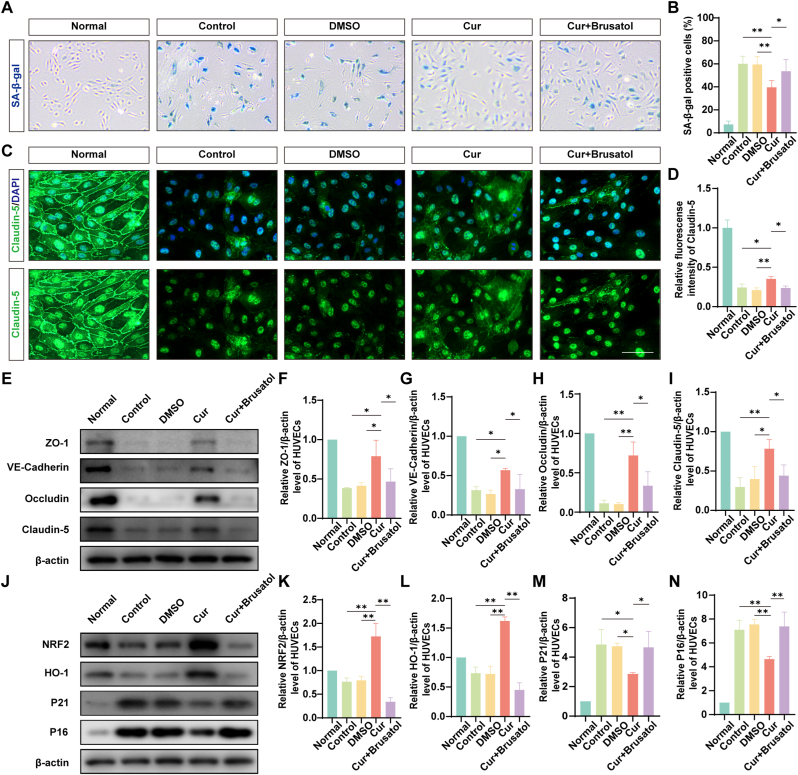
**Reduction in HG/IL-1β-induced senescence markers and TJ protein degradation in HUVECs by Cur treatment.** (A) Typical images of HUVECs stained with SA-β-gal (blue stain indicates senescent cells). The number of SA-β-gal^+^ cells decreased in the Cur group compared to the HG/IL-1β + DMSO group, whereas it increased after brusatol treatment. Scale bar = 200 μm. (B) Quantification of SA-β-gal^+^ cells (n = 5). (C) Typical images of Claudin-5 staining (green, TJ protein). The intensity of Claudin-5 increased in the Cur group compared to the HG/IL-1β + DMSO group, which was decreased by brusatol treatment. Scale bar = 100 μm. (D) Quantification of the relative fluorescence intensity of Claudin-5 (n = 5). (E) Analysis of VE-Cadherin, ZO-1, Occludin and Claudin-5 expression in HUVECs by WB (TJ protein). The level of TJ protein was increased in the Cur group compared to the HG/IL-1β + DMSO group, which was decreased by brusatol treatment. (F–I) Quantification of the level of VE-Cadherin, ZO-1, Claudin-5, Occludin (n = 3). (J) Analysis of NRF2, HO-1, P21 and P16 expression in HUVECs by WB. Note: (1) The levels of NRF2 and HO-1 were upregulated in the Cur group compared to the HG/IL-1β + DMSO group, which was reduced by brusatol treatment. (2) The levels of P21 and P16 were decreased in the Cur group compared to the HG/IL-1β + DMSO group, which were increased by brusatol treatment. (K–N) Quantification of NRF2, HO-1, P21, and P16 levels (n = 3). ∗*P* < 0.05, ∗∗*P* < 0.01. (For interpretation of the references to colour in this figure legend, the reader is referred to the Web version of this article.)

To investigate the therapeutic effects of Cur on HUVECs senescence and its underlying mechanisms, cells were treated with Cur in combination with brusatol, an NRF2 pathway inhibitor. Compared to the HG/IL-1β + DMSO group, SA-β-gal staining indicated a 19.91% ± 5.85% (*p* = 0.0015) reduction in the number of senescent cells in the HG/IL-1β + Cur group, while the reduction in senescent cells induced by Cur was decreased in the presence of brusatol by 13.99% ± 8.33% (*p* = 0.0309) (Fig. 3A and B). Immunofluorescence staining of Claudin-5 showed a 68% increase in intensity (*p* = 0.0036, HG/IL-1β + Cur vs HG/IL-1β + DMSO) and a 49% decrease (*p* = 0.0196, HG/IL-1β + Cur vs HG/IL-1β + Cur + brusatol) (Fig. 3C and D). The changes were also observed by detecting the protein expression of P16 (decreased by 39%, *p* = 0.0026, HG/IL-1β + Cur vs HG/IL-1β + DMSO; increased by 59%, *p* = 0.004, HG/IL-1β + Cur vs HG/IL-1β + Cur + brusatol) and P21 (decreased by 40%, *p* = 0.0406, HG/IL-1β + Cur vs HG/IL-1β + DMSO; increased by 63%, *p* = 0.0495, HG/IL-1β + Cur vs HG/IL-1β + Cur + brusatol) through WB experiment (Fig. 3J). Additionally, Cur treatment enhanced the expression of TJ proteins such as Occludin, ZO-1, VE-Cadherin and Claudin-5, which were otherwise decreased under HG/IL-1β conditions (Fig. 3E–I). Mechanistically, Cur treatment increased the expression of NRF2 and HO-1 by 117% (*p* < 0.0001) and 123% (*p* < 0.0001), respectively, compared to the HG/IL-1β + DMSO group. The expression of NRF2 and HO-1 in the HG/IL-1β + Cur + brusatol group decreased by 80% (*p* < 0.0001) and 72% (*p* < 0.0001), respectively, compared to the HG/IL-1β + Cur group (Fig. 3J).

### 3.4 Preparation and characterization of PM-sEVs

**Fig. 4:**
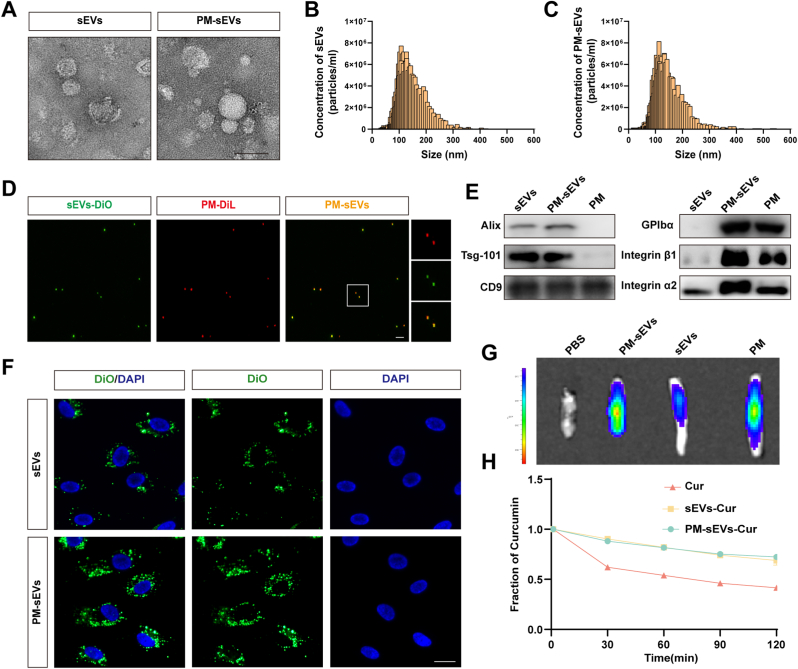
**Preparation and characterization of PM-sEVs.** (A) Typical TEM images of sEVs and PM-sEVs. Both sEVs and PM-sEVs exhibited characteristic cup-shaped morphology. Scale bar = 100 nm. (B, C) Size distribution of sEVs and PM-sEVs by NTA analysis. sEVs and PM-sEVs exhibited similar size distributions. (D) Typical images of PM-sEVs, DiO-labeled sEVs, DiL-labeled PM. Scale bar = 20 μm. The co-localization (yellow signals) of DiO (sEVs) and DiL (PM) fluorescence indicates successful fusion. (E) Analysis of Tsg-101, Alix, CD9, GPIbα, Integrin β1 and Integrin α2 expression of sEVs, PM-sEVs and PM by WB. Note: (1) sEVs markers (Tsg101, Alix, CD9) were enriched in both sEVs and PM-sEVs. (2) PM markers (GPIbα, Integrin β1, Integrin α2) were expressed in PM and PM-sEVs, but absent (or very low) in sEVs, confirming the incorporation of PM proteins onto PM-sEVs. (F) Typical images of DiO-labeled sEVs and PM-sEVs incubated with HUVECs. Scale bar = 20 μm. The significantly enhanced cellular uptake of DiO-PM-sEVs compared to that of DiO-sEVs demonstrated the improved targeting efficiency mediated by the PM coating. (G) IVIS images showing the distribution of sEVs, PM-sEVs and PM in the spinal cord. The fluorescence intensity was higher at the SCI site in the PM-sEVs group than in the sEVs group. (H) Cur release kinetics. The sustained release kinetics of Cur from PM-sEVs-Cur and sEVs-Cur were compared to the faster release of Cur alone. (For interpretation of the references to colour in this figure legend, the reader is referred to the Web version of this article.)

To evaluate the morphology and size distribution of the prepared nanoparticles, transmission electron microscopy (TEM) and nanoparticle tracking analysis (NTA) were used (Fig. 4A–C). NTA analysis revealed average particle sizes of 127.5 nm for sEVs and 135.6 nm for PM-sEVs, while TEM revealed that both sEVs and PM-sEVs exhibited characteristic cup-shaped morphology. To verify successful fusion, DiO-labeled sEVs were fused with DiL-labeled PM. Confocal microscopy revealed colocalization of DiO and DiL signals in the PM-sEVs group (Fig. 4D). sEVs markers (CD9, Alix, and TSG101) were detected in both sEVs and PM-sEVs, while platelet-specific markers (GPIbα, integrin α2, and integrin β1) were observed in PM and PM-sEVs (Fig. 4E) in the WB experiment. The targeting capability of PM-sEVs was evaluated *in vitro* and *in vivo*. *In vitro* experiments showed a higher uptake of PM-sEVs by HUVECs compared to sEVs alone (Fig. 4F). Similarly, in a SCI model, PM-sEVs exhibited stronger fluorescence signals at the lesion site of SCI than sEVs (Fig. 4G). Moreover, the release kinetics of Cur from PM-sEVs-Cur and sEVs-Cur was sustained, in contrast to the faster release from free Cur (Fig. 4H).

### 3.5 PM-sEVs-Cur reduced the expression of senescence markers and TJ protein degradation in HG/IL-1β-treated HUVECs

**Fig. 5:**
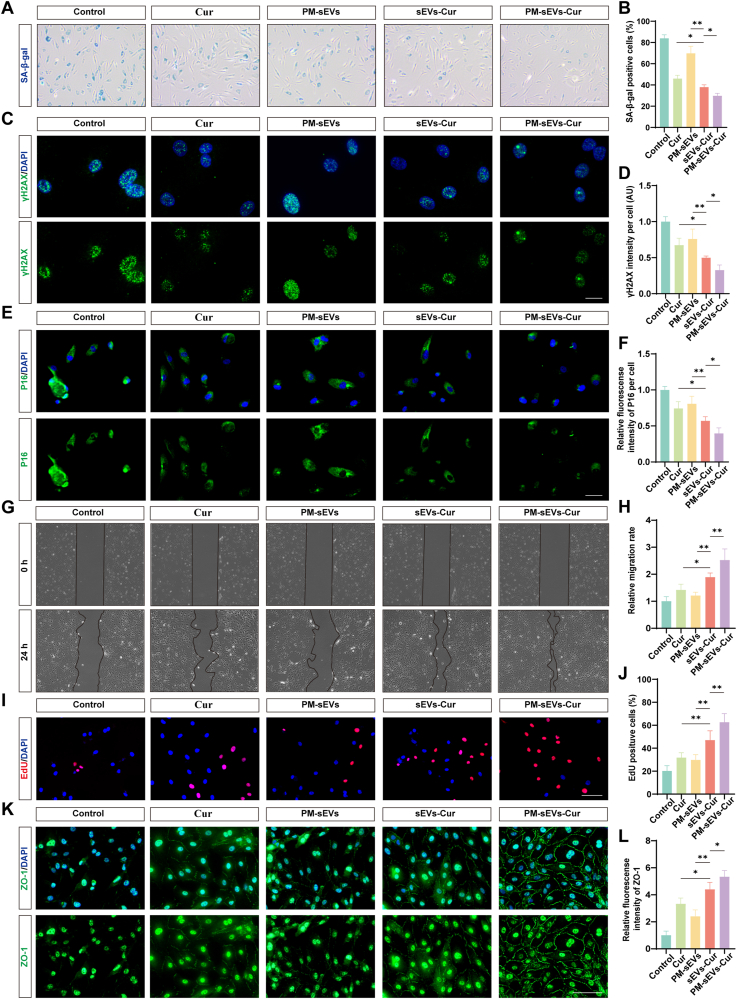
**PM-sEVs-Cur reduced the expression of senescence markers and TJ protein degradation in HG/IL-1β-treated HUVECs.** (A) Typical images of HUVECs stained by SA-β-gal (blue stain indicates senescent cells). The number of SA-β-gal^+^ cells decreased in the PM-sEVs-Cur group. Scale bar = 200 μm. (B) Quantification of SA-β-gal^+^ cells (n = 5). (C) Typical images of HUVECs stained with γH2AX (green puncta within nuclei, a marker of DNA double-strand breaks). The intensity of γH2AX decreased in the PM-sEVs-Cur group. Scale bar = 20 μm. (D) Quantification of γH2AX intensity per cell (n = 5). (E) Typical images of HUVECs stained by P16 (green, senescence marker). The intensity of P16 decreased in the PM-sEVs-Cur group. Scale bar = 50 μm. (F) Quantification of relative fluorescence intensity of P16 per cell (n = 5). (G) Typical images of HUVECs obtained using the scratch assay. PM-sEVs-Cur treatment accelerated wound closure. (H) Quantification of migration rate at 24h (n = 5). (I) Typical images of EdU-stained HUVECs (red, proliferation marker). The number of EdU^+^ cells increased in the PM-sEVs-Cur group. Scale bar = 100 μm. (J) Quantification of EdU^+^ cells (n = 5). (K) Typical images of HUVECs stained with ZO-1 (green, tight junction protein). The intensity of ZO-1 increased in the PM-sEVs-Cur group. Scale bar = 100 μm. (L) Quantification of the relative fluorescence intensity of ZO-1 (n = 5). ∗*P* < 0.05, ∗∗*P* < 0.01. (For interpretation of the references to colour in this figure legend, the reader is referred to the Web version of this article.)

The ability of PM-sEVs-Cur to mitigate HG/IL-1β-induced HUVECs senescence *in vitro* was assessed. SA-β-gal staining showed that the sEVs-Cur group had 8.08% ± 2.65% (*p* = 0.0058) fewer senescent cells than the Cur group, with the PM-sEVs-Cur group exhibiting the most pronounced reduction of 16.27% ± 4.56% (*p* = 0.0046) (Fig. 5A and B). Immunofluorescence staining showed a 35% reduction in γH2AX fluorescence intensity (*p* = 0.04) and a 30% reduction in P16 fluorescence intensity (*p* = 0.0181) in the PM-sEVs-Cur group compared to the sEVs-Cur group (Fig. 5C–F). As shown by the scratch assay and EdU staining, the PM-sEVs-Cur group enhanced cell migration and proliferation (Fig. 5G–J). Compared with the Cur group and the sEVs-Cur group, the percentage of EdU^+^ cells increased by 30.77% ± 9.57% (*p* < 0.0001) and 15.59% ± 3.51% (*p* = 0.0046) in the PM-sEVs-Cur group. Additionally, the beneficial effects of PM-sEVs-Cur extended to the restoration of TJ protein expression. Immunofluorescence staining showed that the PM-sEVs-Cur group resulted in 60% less degradation of ZO-1 protein than the Cur group (*p* < 0.0001) and 21% less degradation than the sEVs-Cur group (*p* = 0.0261) (Fig. 5K and L).

### 3.6 PM-sEVs-Cur decreased endothelial senescence and BSCB leakage in DM-SCI

**Fig. 6:**
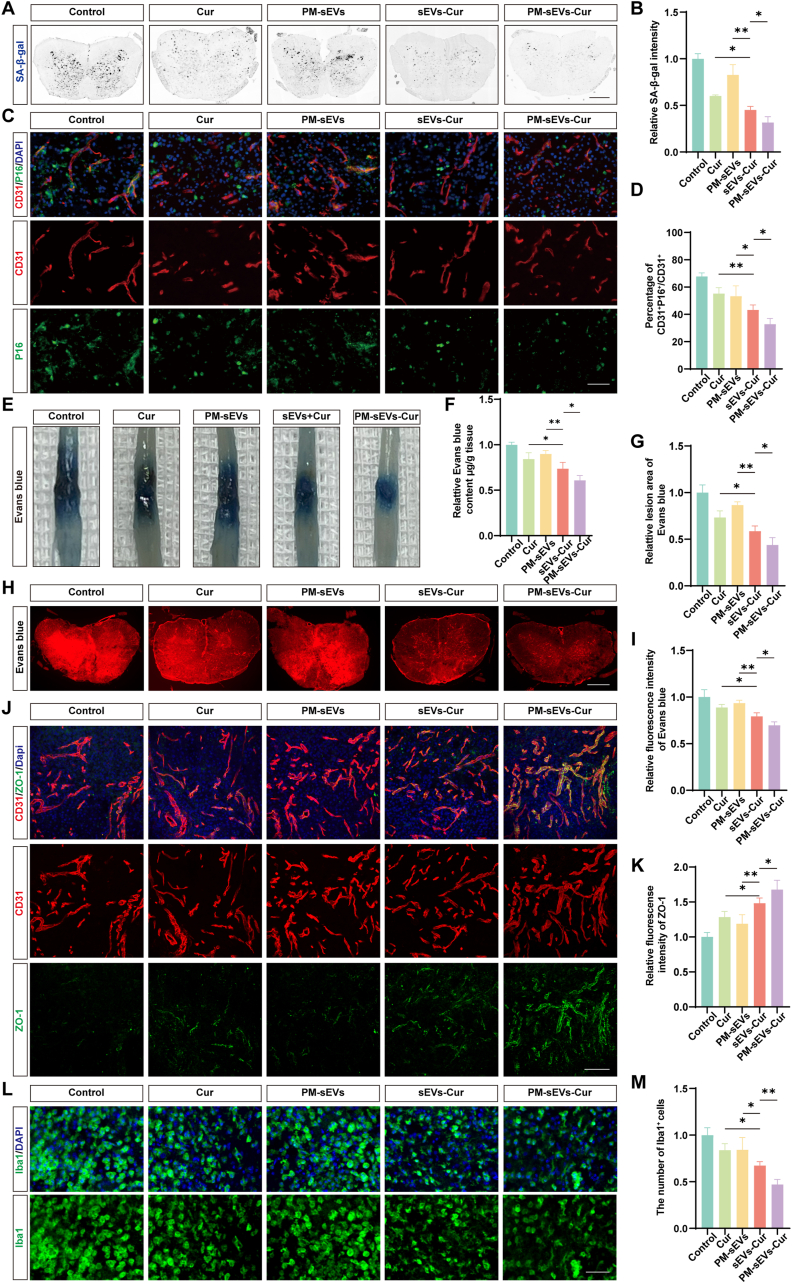
**PM-sEVs-Cur decreased endothelial senescence and BSCB leakage in DM-SCI.** (A) Typical images of spinal cord stained with SA-β-gal (black precipitate indicates senescent cells). Treatment with PM-sEVs-Cur decreased the number of senescent cells. Scale bar = 400 μm. (B) Quantification of the relative intensity of SA-β-gal (n = 5). (C) Typical images of spinal cord stained with CD31 and P16. The merged image showed co-localization (yellow), indicating senescent ECs. Treatment with PM-sEVs-Cur decreased the number of senescent ECs. Scale bar = 100 μm. (D) Quantification of percentage of CD31^+^P16^+^/CD31^+^ cells (n = 5). (E) Typical images of Evans Blue leakage test. Visual inspection showed more intense and widespread blue staining in the Control group than in the Cur group, indicating greater leakage, which was diminished by PM-sEVs-Cur treatment. (F) Quantification of the relative content of Evans Blue (n = 5). (G) Quantification of relative lesion area (n = 5). (H) Typical red fluorescence images of Evans Blue extravasation. The decreased red fluorescence intensity in the PM-sEVs-Cur group compared to that in the control group demonstrated the protection of BSCB integrity. Scale bar = 400 μm. (I) Quantification of the relative fluorescence intensity of Evans Blue (n = 5). (J) Typical images of spinal cord stained by CD31 (red) and ZO-1 (green). The Control group exhibited a visible reduction and discontinuity of ZO-1 staining along CD31^+^ vessels, which was restored by PM-sEVs-Cur. Scale bar = 100 μm. (K) Quantification of the relative fluorescence intensity of ZO-1 (n = 5). (L) Typical images of spinal cord stained with Iba1 (green, microglia/macrophages marker). Treatment with PM-sEVs-Cur decreased the number of Iba1^+^ cells in the lesion site. Scale bar = 50 μm. (M) Quantification of the number of Iba1 (n = 5). ∗*P* < 0.05, ∗∗*P* < 0.01. (For interpretation of the references to colour in this figure legend, the reader is referred to the Web version of this article.)

The therapeutic potential of PM-sEVs-Cur was further evaluated in a DM-SCI model. SA-β-gal staining at 7 days after SCI revealed that PM-sEVs-Cur treatment reduced the number of senescent cells by 47% compared to the Cur group (p < 0.0001), and by 30% compared to the sEVs-Cur group (p = 0.0257) (Fig. 6A and B). The superior efficacy of PM-sEVs-Cur was further verified by immunofluorescence analysis of P16 and CD31 co-expression (Fig. 6C and D). The lowest percentage of P16^+^ CD31^+^ cells was found in the PM-sEVs-Cur group (32.79% ± 4.14%), followed by the sEVs-Cur group (43.23% ± 3.65%) and the Cur group (51.19% ± 4.36%). To assess BSCB integrity, the PM-sEVs-Cur group exhibited a 17% reduction in Evans Blue dye accumulation (p = 0.0101) and a 12% reduction in fluorescence intensity (p = 0.0316) compared to the sEVs-Cur group (Fig. 6E–I). Immunofluorescence staining of ZO-1 corroborated these findings, showing the highest relative fluorescence intensity of ZO-1 in the PM-sEVs-Cur group (1.68 ± 0.12) compared to the other groups (Fig. 6J and K). Furthermore, PM-sEVs-Cur reduced> inflammation, as evidenced by a 30% (*p* = 0.0068) decrease in the number of Iba1^+^ cells aggregating in the lesion site (Fig. 6L and M).

### 3.7 PM-sEVs-Cur enhanced the BMS score and reduced the lesion area in DM-SCI

**Fig. 7:**
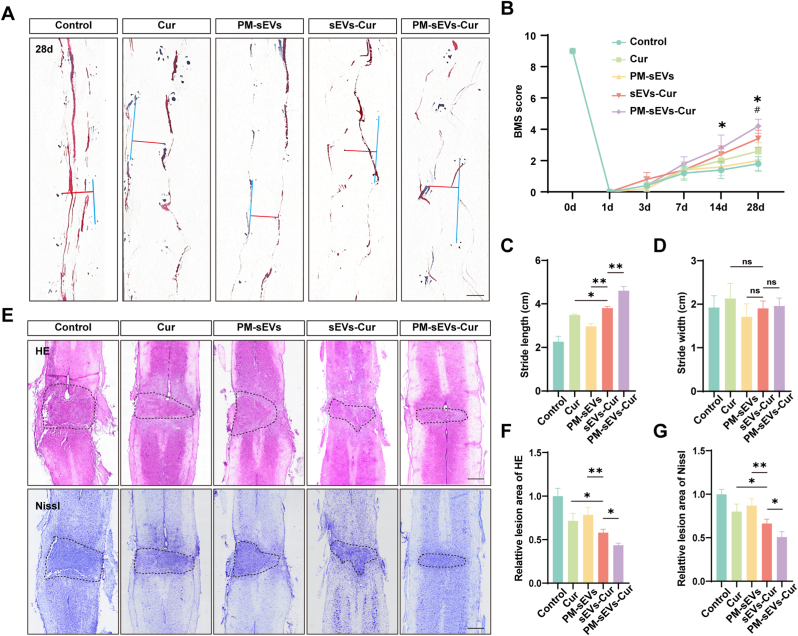
**PM-sEVs-Cur enhanced the BMS score and reduced the lesion area in DM-SCI.** (A) Typical images of footprint (blue inkpad for forelimbs and red inkpad for hindlimbs). The PM-sEVs-Cur group showed improved coordination and reduced dragging compared to the Control group. Scale bar = 1 cm. (B) BMS scores at different time points (n = 5). (C) Quantitative analysis of stride length (n = 5). (D) Quantitative analysis of stride width (n = 5). (E) Typical images of spinal cord stained by HE and Nissl. PM-sEVs-Cur treatment reduced the lesion area compared to that in the Control group. Scale bar = 500 μm. (F, G) Quantitative assessment of the lesion area in the spinal cord by HE and Nissl staining (n = 5). # sEVs-Cur group compared with Cur group; ∗ PM-sEVs-Cur group compared with sEVs-Cur group. ∗*P* < 0.05, ∗∗*P* < 0.01; ns indicates not significant. (For interpretation of the references to colour in this figure legend, the reader is referred to the Web version of this article.)

Motor function recovery in DM-SCI was evaluated using motor footprints and BMS score. The fact that mice had BMS score of 0 after modeling indicated that the SCI model was successfully established. No significant differences were observed in the BMS scores of mice during the initial 7 days following SCI. However, the PM-sEVs-Cur group exhibited the highest BMS score at 14 days (3.2 ± 0.45) and 28 days (4.2 ± 0.45) after SCI (Fig. 7B). For a more visual analysis of the motor function recovery, the motor footprints of mice were recorded and analyzed at 28 days after SCI (Fig. 7A). No significant differences were observed in step width among the groups (Fig. 7D). However, compared with the Cur group, the step length was longer in the sEVs-Cur group by 9% (*p* = 0.033) and the PM-sEVs-Cur group by 32% (*p* < 0.0001). The PM-sEVs-Cur group exhibited the longest step length (4.47 ± 0.33) (Fig. 7C). Nissl and HE staining respectively showed 25% (*p* = 0.0132) and 24% (*p* = 0.0296) reductions in lesion area in the PM-sEVs-Cur group compared with the sEVs-Cur group (Fig. 7E–G). Furthermore, histological examination of major organs (liver, heart, spleen, kidney, lung) at 28 days post-SCI indicated no apparent treatment-related toxicity for PM-sEVs-Cur (Fig. S1).

### 3.8 PM-sEVs-Cur decreased neuronal loss and increased axonal regeneration in DM-SCI

**Fig. 8:**
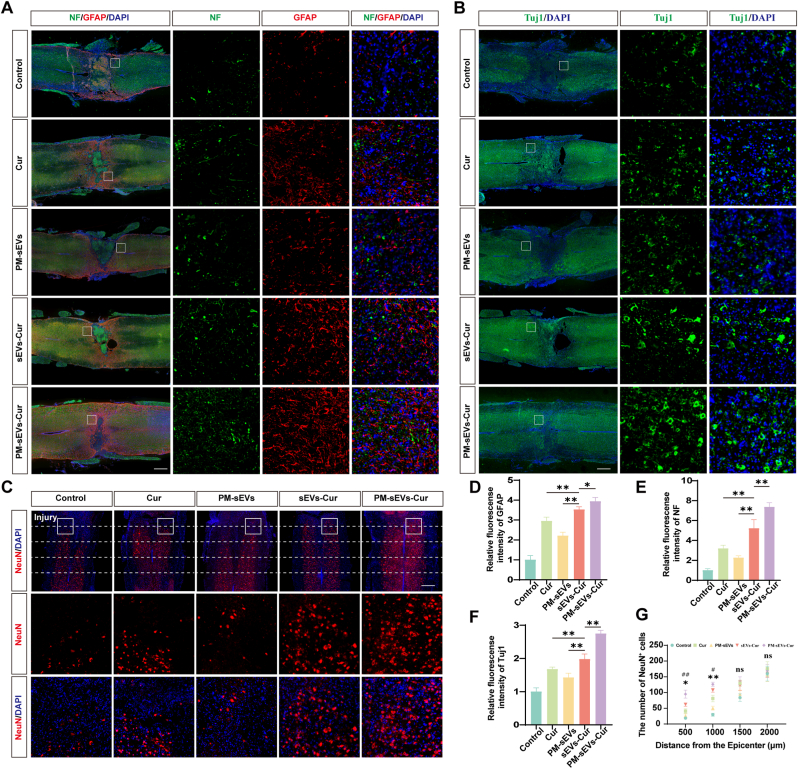
**PM-sEVs-Cur decreased neuronal loss and increased axonal regeneration in DM-SCI.** (A) Typical images of spinal cord stained by NF (green, axonal marker) and GFAP (red, astrocyte marker). Treatment with PM-sEVs-Cur increased the intensity of NF and GFAP at the vicinity of the lesion area. Scale bar = 500 μm. (B) Typical images of spinal cord stained with Tuj1 (green**,** neuronal/axonal marker). Treatment with PM-sEVs-Cur enhanced Tuj1 signal intensity surrounding the lesion area. Scale bar = 500 μm. (C) Typical images of spinal cord stained with NeuN (mature neuronal marker). Treatment with PM-sEVs-Cur increased the number of NeuN^+^ neurons compared to sEVs-Cur treatment adjacent to the lesion area. Scale bar = 500 μm. (D) Quantification of the relative fluorescence intensity of GFAP (n = 5). (E) Quantification of the relative fluorescence intensity of NF (n = 5). (F) Quantification of the relative fluorescence intensity of Tuj1 (n = 5). (G) Quantification of NeuN^+^ cells at different distances from the lesion core (n = 5). # sEVs-Cur group compared with Cur group; ∗ PM-sEVs-Cur group compared with sEVs-Cur group. ∗*P* < 0.05, ∗∗*P* < 0.01; ns indicates not significant. (For interpretation of the references to colour in this figure legend, the reader is referred to the Web version of this article.)

To further explore the mechanisms underlying the promotion of motor function recovery in DM-SCI by PM-sEVs-Cur, immunofluorescence staining of the spinal cord was conducted at 28 days after SCI. Immunofluorescence analysis revealed a higher fluorescence intensity of NF and GFAP in the peri-lesional area by 41% (*p* < 0.0001) and 12% (*p* = 0.0186), respectively, in the PM-sEVs-Cur group than in the sEVs-Cur group (Fig. 8A). A similar result was also observed in the immunofluorescence staining of Tuj1 by 38% (*p* < 0.0001) (Fig. 8B). Subsequently, immunofluorescence staining of NeuN was performed, and the number of NeuN^+^ cells at different distances from the center of the injury area was counted (Fig. 8C). At 500 μm distal to the injury site, the number of NeuN^+^ cells in the PM-sEVs-Cur group (95 ± 12.7) was the highest compared with that in the Cur group (41.2 ± 7.12, *p* = 0.0008) and the sEVs-Cur group (62 ± 5.43, *p* = 0.0131).

## 4 Discussion

Advanced age and diabetes cause the pathological accumulation of senescent cells in chronic wounds, maintained by pro-inflammatory secretions and senescence-associated secretory phenotype (SASP), glycation end-products in advanced stage, and oxidative damage [50]. To date, few studies have focused on the increased number of senescent cells after SCI, particularly with a focus on ECs [9,51]. Previous research showed that SCI induced cellular senescence, primarily in neuronal cells [9]. However, emerging evidence has demonstrated that SCI triggers ECs senescence accompanied by SASP development. This pathological cascade critically regulates neuroinflammatory responses and impairs neural functional recovery, thereby establishing ECs senescence as a key mediator of secondary injury progression post-SCI [51]. As reported, hyperglycemia causes premature senescence in ECs [6,8]. Consistent with our experimental findings, senescent cells accumulate in neurons and ECs following SCI. The proportion of senescent ECs was about 26.27% ± 6.07% in the DM-Sham group. And it was increased in the DM-SCI group compared to the SCI group by approximately 18.87% ± 5.91%, suggesting that the pre-existing diabetes potentiated SCI-induced ECs senescence. The barrier comprised by ECs is an important factor in protecting the spinal parenchyma from toxic substances and disruption of spinal homeostasis [[52], [53], [54]]. However, Evans Blue extravasation assays showed that the permeability of the BSCB increased by 16% in the DM-SCI group compared to that in the SCI group. These observations in DM-SCI mice demonstrated that diabetes exacerbated ECs senescence and BSCB leakage post-SCI, underscoring the detrimental role of senescent ECs in SCI and suggesting innovative therapeutic targets for the treatment of DM-SCI.

Cur exhibits therapeutic potential across a broad spectrum of diseases, including its well-documented anti-senescence properties [55]. Experimental data demonstrated that Cur treatment significantly reduced HG/IL-1β-induced senescence in HUVECs by 19.91% ± 5.85%. Mechanistically, Cur has been shown to induce antioxidant protective genes through NRF2/HO-1 pathways. NRF2 is the main regulator of cellular responses to environmental stresses [22]. Previous studies have reported that Cur could prevent oxidative stress-induced cellular senescence via the NRF2/HO-1 pathway in cochlear hair cells [56]. This aligns with our data, which showed that Cur enhanced the upregulated expression of NRF2 and HO-1 in HUVECs treated with HG/IL-1β, which was suppressed by brusatol. Senescent cells induced by SCI display multiple senescent characteristics. These include the co-expression of SA-β-gal along with P21 or P16 and γH2AX. Additionally, there is a decline expression of BrdU [9]. Similarly, in our experiments, HUVECs also showed changes of senescent characteristics induced by HG/IL-1β. Through Cur treatment, the senescent characteristics of ECs were improved, while the therapeutic effect was significantly inhibited by brusatol. As SA-β-gal staining indicated that the number of senescent cells increased by 13.99% ± 8.33% in the presence of brusatol compared to Cur treatment alone. When senescent characteristics were improved, the expression of TJ, NRF2, and HO-1 showed an upward trend. However, when Cur and brusatol were used simultaneously, the therapeutic effect of Cur was diminished. As shown in the immunofluorescence staining of Claudin-5, the intensity decreased by 49% in the HG/IL-1β + Cur + brusatol group compared to the HG/IL-1β + Cur group. Taken together, these data demonstrated that Cur was able to attenuate HG/IL-1β-induced HUVECs senescence and TJ protein degradation through the NRF2/HO-1 pathway. However, it should be pointed out that Cur has a poor water solubility. This results in low bioavailability of Cur in animal experiments. Consequently, choosing an appropriate carrier is crucial and of great significance.

Although the drug delivery of stem cell-derived sEVs is promising, the problems of limited yields and targeting are urgent issues that need to be solved [37,57,58]. In contrast, milk-derived exosomes have been shown to provide notably higher yields, offering a potential solution to the challenges of scalability [59]. In the study, we successfully extracted milk-derived sEVs and loaded them with Cur. Histological analysis revealed no systemic toxicity. sEVs exhibit advantages such as cross-species biocompatibility, the potential to traverse the BBB, and the function of acting as a delivery vehicle for both lipophilic and hydrophilic macromolecules [60]. *In vivo* experiments demonstrated that milk-derived sEVs successfully traversed the BSCB and localized to the SCI lesion. Platelets inherently possess the capacity to migrate towards the vascular wall and precisely target sites of vascular injury. For this purpose, nanoparticles mimicking platelets have been developed to fulfill hemostatic functions and enable targeted delivery [30,32]. However, no studies have reported its targeted therapeutic effects in SCI. When PM were successfully isolated and fused with sEVs, PM-sEVs-Cur maintained the original characteristics of sEVs and increased the stability of Cur in our study. sEVs are naturally occurring nanoscale membrane particles (50–150 nm) [24]. Our experiments demonstrated that the isolated sEVs and PM-sEVs exhibited the diameters of 127.5 nm and 135.6 nm, respectively, and displayed cup-shaped morphology under electron microscopy. Western blot analysis further revealed that PM-sEVs expressed marker proteins characteristic of both PM and sEVs. These results indicate that PM-sEVs were successfully fabricated. Moreover, compared with unmodified sEVs, PM-sEVs showed superior targeting in the lesion of SCI.

*In vitro*, the treatment of PM-sEVs-Cur reduced the number of senescent cells by approximately 16.27% ± 4.56% and mitigated the degradation of ZO-1 protein by 60% compared to the treatment of Cur alone. These results showed that PM-sEVs-Cur had an outstanding ability to mitigate the HG/IL-1β-induced HUVECs senescence and TJ protein degradation. *In vivo*, PM-sEVs-Cur (32.79% ± 4.14%) also demonstrated superior efficacy compared to Cur alone (51.19% ± 4.36%) or sEVs-Cur (43.23% ± 3.65%) in mitigating ECs senescence, thereby enhancing the protection of BSCB integrity. Furthermore, PM-sEVs-Cur promoted axon regeneration (increased by 41%, vs sEVs-Cur), reduced neuronal loss (decreased by 53%, vs sEVs-Cur), and improved motor function recovery (increased by 17% of BMS score, vs sEVs-Cur) in DM-SCI. These findings demonstrate that PM-mediated targeted delivery significantly enhances the therapeutic efficacy of sEVs-Cur in mitigating DM-SCI pathology and promoting recovery.

Despite these promising results, several limitations should be considered. First, although permeability assays that depend on cultured monolayers of ECs are often utilized, they may not completely embody the physiological characteristics of the BSCB. Consequently, the co-culture of ECs and astrocytes may be a more appropriate model for future research. Second, while our study primarily focused on the NRF2/HO-1 pathway, there may be other mechanisms through which Cur acts in DM-SCI recovery, which need further investigation. Third, although PM-sEVs-Cur targeted ECs, the release of Cur may also affect other cells, which needs to be explored in subsequent research.

## 5 Conclusion

This study demonstrated the therapeutic potential of PM-sEVs-Cur for targeted delivery in DM-SCI. Diabetes exacerbated ECs senescence and BSCB disruption following SCI. In vitro experiments showed that Cur effectively mitigated HG/IL-1β-induced HUVECs senescence through activation of the NRF2/HO-1 pathway. PM-sEVs-Cur successfully localized to the SCI lesion, attenuated ECs senescence, preserved BSCB integrity, and promoted axonal regeneration and motor function recovery in DM-SCI. These findings highlight PM-sEVs-Cur as a promising targeted therapeutic delivery platform for the treatment of DM-SCI.

## CRediT authorship contribution statement

**Yaozhi He:** Writing – review & editing, Writing – original draft, Validation, Methodology, Investigation, Formal analysis, Data curation, Conceptualization. **Siyuan Zou:** Validation, Methodology, Formal analysis, Data curation. **Jiawei Wang:** Validation, Methodology, Formal analysis, Data curation. **Wenbin Zhang:** Methodology. **Sheng Lu:** Methodology. **Mengxian Jia:** Methodology. **Yumin Wu:** Validation. **Xiaowu Lin:** Validation. **Ziwei Fan:** Investigation. **Qishun Liang:** Investigation. **Yizhe Sheng:** Investigation. **Qichuan Zhuge:** Investigation. **Bi Chen:** Supervision, Resources, Funding acquisition, Conceptualization. **Minyu Zhu:** Supervision, Resources, Funding acquisition, Conceptualization. **Honglin Teng:** Writing – review & editing, Supervision, Funding acquisition, Data curation, Conceptualization.

## Declaration of competing interest

The authors declare no competing financial interest.

## Figures and Tables

**figs1:**
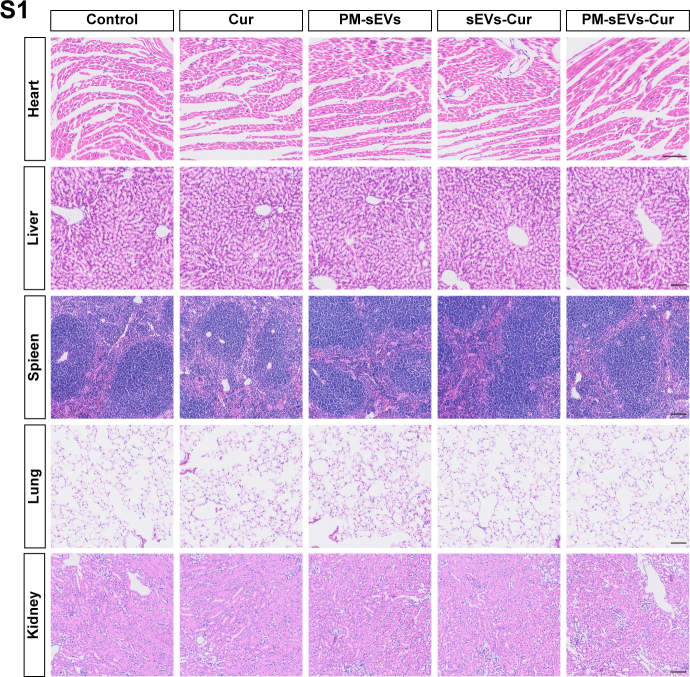


**figs2:**
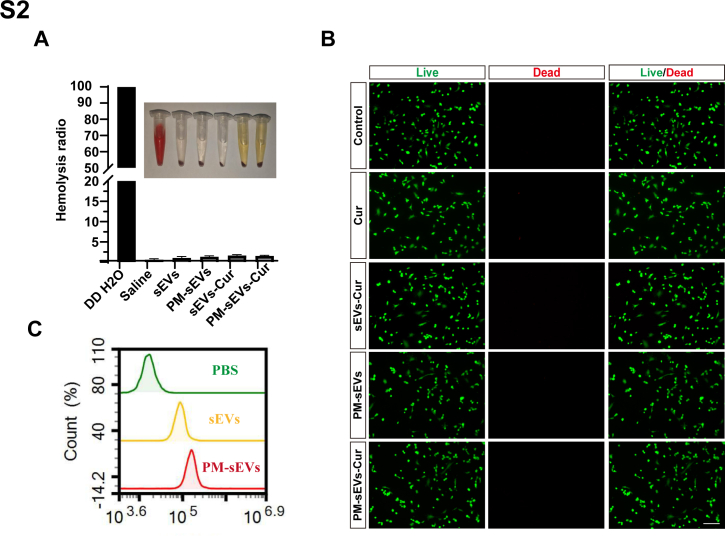


## Data Availability

Data will be made available on request.
